# The Effect of Conventional Ho:YAG, Magneto Ho:YAG and Thulium Fiber Laser on Soft Tissue: An Ex Vivo Comparative Study in Porcine Kidney

**DOI:** 10.3390/jcm15031074

**Published:** 2026-01-29

**Authors:** Theodoros Spinos, Dimitra Gkanetsou, Vasileios Tatanis, Angelis Peteinaris, Michail Papapanou, Moisés Rodríguez Socarrás, Fernando Gómez Sancha, Athanasios Vagionis, Georgios-Eleftherios Anagnostopoulos, Evangelos Liatsikos, Panagiotis Kallidonis

**Affiliations:** 1 Department of Urology, University of Patras Hospital, 26504 Patras, Greecetatanisbas@gmail.com (V.T.);; 2 Department of Pathology, University of Patras Hospital, 26504 Patras, Greece; 3 Second Department of Obstetrics and Gynecology, Aretaieion University Hospital, National and Kapodistrian University of Athens, 11528 Athens, Greece; 4 Department of Urology and Robotic Surgery, Instituto de Cirugía Urológica Avanzada (ICUA)-Clínica CEMTRO, 28035 Madrid, Spain; 5 Department of Urology, Medical University of Vienna, 1090 Vienna, Austria

**Keywords:** endourology, lasers, Magneto, Ho:YAG, TFL, soft tissue

## Abstract

**Background/Objectives**: In an attempt to combine the benefits of the Holmium:YAG (Ho:YAG) laser and Thulium Fiber Laser (TFL), the “Magneto” mode lowers the peak power of the Ho:YAG laser, generating longer duration pulses. The purpose of this study is to compare the effect of the standard virtual basket (VB) Ho:YAG laser, Magneto Ho:YAG laser and TFL on soft tissue in an ex vivo model. **Methods**: Two renal units from a female pig were used for the current experiment. Sixteen distinct areas were defined. Each area included three parallel lines, which were made with the three different laser technologies. The VB Ho:YAG laser was used for the first line and the Ho:YAG laser in the “Magneto mode” was used to generate the second line, while the third line was performed with a TFL in short pulse mode. The same laser settings (1 J/10 Hz/10 W) and the same fiber diameter (200 μm) were used for all three laser incisions. The same surgeon performed all incisions with a standardized and repeatable technique, controlling hand speed and distance of laser fiber from kidney surface using the stabilization setup. Sections of the selected areas produced distinct paraffin blocks, each one containing three parallel laser lines. Two independent pathologists evaluated the incision depth, incision width, coagulation depth and carbonization effect of the three different lasers. **Results**: Although the incision depth and the carbonization effect were comparable between the three lasers, incision width and coagulation depth showed a statistically significant difference. Median incision width was 1.17 (1.04, 1.99) mm for the VB Ho:YAG laser, 1.05 (0.89, 1.50) mm for the Magneto Ho:YAG laser and 0.82 (0.65, 0.88) mm for the TFL (*p* = 0.001). The coagulation depth was 0.49 (0.41, 0.56) mm for the VB Ho:YAG laser, 0.51 (0.39, 0.59) mm for the Magneto Ho:YAG laser and 0.18 (0.17, 0.23) mm for the TFL (*p* < 0.001). During post hoc analysis for the three comparisons, the differences between the VB Ho:YAG laser and TFL and between the Magneto Ho:YAG laser and TFL were statistically significant for both parameters. **Conclusions**: Both the VB and Magneto Ho:YAG lasers produced laser incisions with statistically significant greater incision width and coagulation depth than the TFL on the ex vivo model. Overall, the Magneto Ho:YAG laser was associated with the greatest median coagulation depth. Post Hoc Man–Whitney tests for the three comparisons revealed statistically significant differences only between the VB Ho:YAG laser and TFL and between the Magneto Ho:YAG laser and TFL. This finding could potentially be translated into better haemostasis during endourological soft tissue surgery. The implementation of additional studies, both experimental and clinical ones, is of outmost importance to draw safer conclusions.

## 1. Introduction

Lasers have totally revolutionized the endourological management of urolithiasis, benign prostatic obstruction (BPO) and upper tract urothelial carcinoma (UTUC), while they are also very useful tools for the management of non-muscle invasive bladder cancer (NMIBC) and benign conditions, such as ureteropelvic junction obstruction (UPJO) and strictures [1,2,3]. It thus becomes clear that lasers have multiple applications on soft tissue endourological surgeries [4,5,6]. The Holmium:YAG (Ho:YAG) laser is a solid-state pulsed laser with a wavelength of 2100 nm, which undoubtedly represents the most studied laser in Urology [3,7]. Moreover, the introduction of the Holmium Laser Enucleation of the Prostate (HoLEP) technique by Gilling et al. led to further popularization of its use in Endourology [8,9]. Recently, evolution of the Ho:YAG laser technology, including the production of high-power generators and invention of pulse modulation, expanded its applications and optimized its outcomes [10,11]. However, over the last few years, Endourologists also witnessed the advent of the Thulium Fiber Laser (TFL), which presents a different wavelength of 1940 nm, higher water absorption coefficient, lower peak power and smaller diameters of operating fibers [12,13]. The TFL has gained increased popularity due to its excellent dusting capabilities and its ability to produce precise cuts with reduced tissue damage, currently being the laser of choice by many Urologists [12,13]. In an attempt to combine the advantages of both the high-power Ho:YAG laser and the TFL, Quanta System developed, in its latest Cyber Ho generator, a new innovative technology, called “Magneto”. The Magneto mode enables a reduction in the peak power of the Ho:YAG laser down to 500 W. In this manner, it simulates the physical properties of the TFL, producing longer duration pulses. On the contrary, the peak power of the conventional Ho:YAG generators ranges from 2000 W to 10,000 W [14]. Although the Magneto mode is obviously associated with better dusting capabilities and lower retropulsion during endourological stone disease management, its potential benefits during endourological soft tissue procedures have not yet been investigated [14]. Moreover, existing studies focus on lithotripsy [15,16,17], while its applications in soft tissue surgery are limited [14]. The TFL’s physical properties have been associated with improved haemostasis in some studies and thus we can hypothesize that Magneto’s lower peak power could potentially replicate these results [18]. The purpose of this experiment is to compare the effect of the standard Ho:YAG laser, Magneto Ho:YAG laser and TFL on soft tissue in an ex vivo model. The ablative and coagulative profiles of the three different laser technologies were investigated, comparing their incision depth, incision width, coagulation depth and carbonization effect on soft tissue.

## 2. Materials and Methods

### 2.1. Animal Model

An ex vivo experimental study was performed in order to assess the different effects of the three lasers on soft tissue. A dedicated study protocol was carefully designed in line with the declaration of Helsinki and all its later amendments. Ethical approval was given by both the Veterinary Administration of the Prefecture of Western Greece and the Ethics Committee of University of Patras. Two renal units from a single female pig, weighing 30 kg, were used for the current experiment, in accordance with the Principles of Ethics in Animal Experimentation [19]. Taking into consideration that this is a feasibility study, only one pig was used for ethical reasons. However, both surfaces of the two kidneys were utilized in order to perform several incisions and allow repeatability. In an attempt to achieve optimal surgical conditions, the pig was nil per os 12 h before the experiment. Before intubation, the pig was sedated using intramuscular injections of ketamine (5 mg/kg) and xylazine (1 mg/kg). The left ear vein was utilized for venous access and propofol (5%) was used for general anesthesia. The pig was then intubated and placed in supine position. Paracetamol was administered intravenously in order to provide analgesia during the experiment. Afterwards, radical nephrectomy under general anesthesia was performed on both sides, by two experienced surgeons. The operation included a midline vertical incision, followed by mobilization of the intestines in order to expose and then dissect the two kidneys. The two ureters were then clipped, while both renal pedicles were ligated with sutures and clips. After finishing the extraction of the two kidneys, all the layers were sutured. At the end of the procedure, the animal was sacrificed. The ARRIVE Checklist for animal studies is presented as Supplementary Material S1.

### 2.2. Experimental Setup and Laser Settings

**Figure 1:**
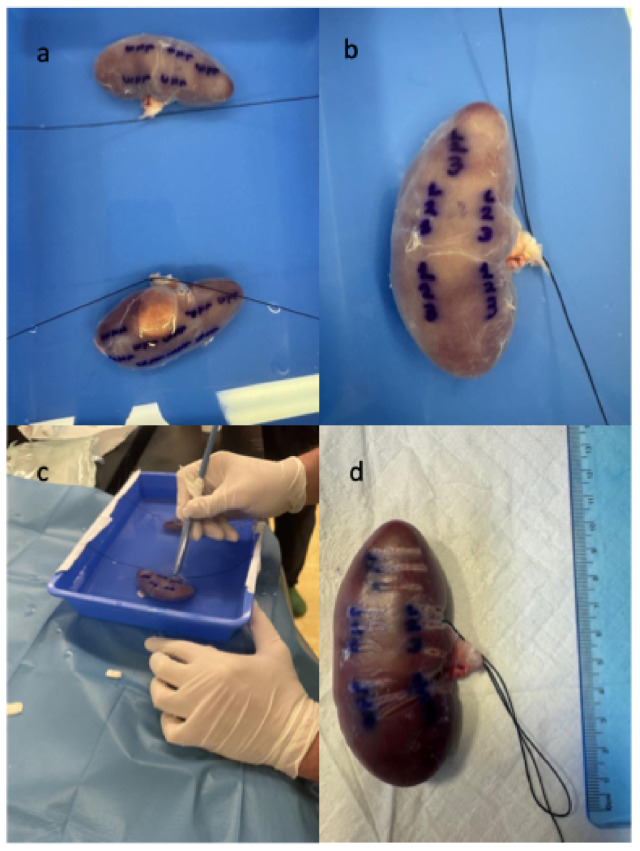
(**a**) The stabilization of the two porcine kidneys, (**b**) the marked regions, (**c**) generation of laser incisions with different lasers, and (**d**) the three parallel lines indicating different laser technologies.

**Figure 2:**
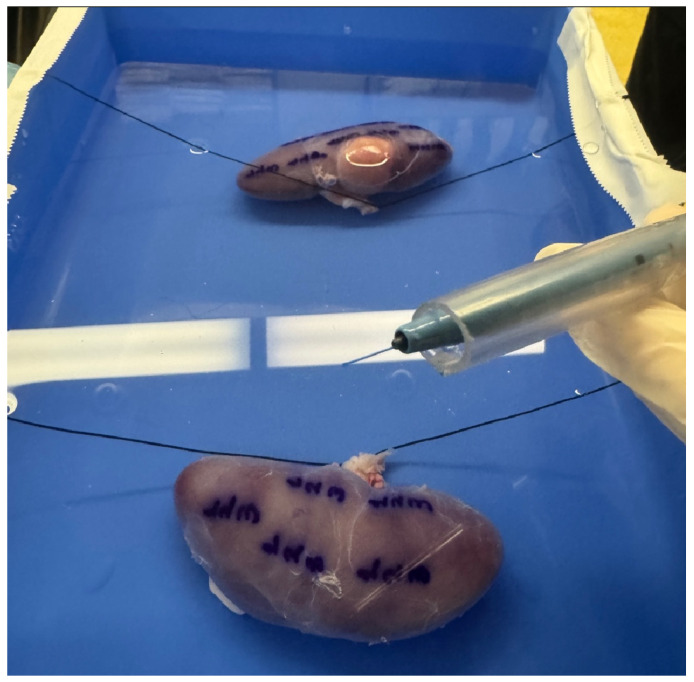
For the experimental setup, the fiber was secured within a 16 Fr Amplatz dilator (Cook Medical) placed inside a glass cylinder.

All steps of the experiment were performed as fast as possible in an attempt to achieve a maximum interval of 30 min between tissue fixation and ligation of the two renal pedicles. We used the limit of 30 min, based on studies on partial nephrectomies showing that after 30 min of warm ischemia time, renal dysfunction can result [16]. After extraction of the two kidneys, the perinephric fat was removed. The two kidneys were fixed in a plastic tank filled with normal saline, using Vicryl sutures (2-0) and glue pads (UHU patafix) (Figure 1a). The water level was at least ~10 mm above the kidney surface. Both surfaces of the two kidneys were utilized in order to define sixteen distinct areas, which were marked with the numbers 1, 2 and 3, using a blue permanent marker. Each number corresponded to a distinct laser technology and then three parallel incisions with the three lasers were performed starting from each number (Figure 1c,d). The conventional Ho:YAG laser (Cyber Ho Magneto 150 W) in virtual basket (VB) soft tissue mode was used for the first line. The same Ho:YAG generator in “Magneto mode” was used to generate the second line, while the third line was performed with the TFL (EMS LaserClast) in custom ablation mode, with a short pulse setting for uniformity reasons. The same laser settings (1 J/10 Hz/10 W) and the same fiber diameter (200 μm) were used for all three laser incisions. A glass cylinder and a 16 Fr Amplatz percutaneous nephrolithotomy dilator (Cook Medical) were used to secure the laser fiber and ensure efficient grip (Figure 2). The same surgeon performed all incisions with a standardized and repeatable technique, controlling hand speed and distance of laser fiber from kidney surface using the stabilization setup. All incisions were performed within a predefined duration of three seconds, while the surgeon’s hand was also stabilized on the water-filled tank. After the implementation of the experiment, all incisions (3 incisions for each marked area, 48 in total) were carefully reviewed for uniformity. The two porcine kidneys were then transferred to the Department of Pathology, for subsequent histopathologic analysis.

### 2.3. Histopathological Assessment

Only 12 out of the 16 initially marked areas were included in final analysis, corresponding to the ones with the most uniform lines. This step was followed in an attempt to eliminate the error of manual movement. Lines with different lengths or in close proximity were excluded, and this decision was blinded to the type of laser used. Sections of the 12 selected areas produced 12 distinct paraffin blocks, each one containing three parallel laser lines. Three slides from each block (36 in total) were evaluated, representing different incision depths. This strategy was followed in an attempt to eliminate potential differences between incisions, arising from manual movement of the laser fiber. All slides were then stained with hematoxylin–eosin (H&E) and Masson’s trichrome. Two independent pathologists evaluated the incision depth, incision width, coagulation depth and carbonization effect of the three different lasers. The two pathologists were blinded and did not know which line corresponded to each laser technology. The coagulation depth was defined as the vertical distance from the renal surface to the deepest clotted blood vessel that could be spotted by the pathologist. A predefined three-grade scale was used to evaluate the carbonization effect: grade 1 (0–0.128 mm^2^), grade 2 (0.128–0.445 mm^2^) and grade 3 (>0.445 mm^2^).

### 2.4. Statistical Analysis

The Stata statistical software (version 15.0) was used for statistical analysis [20]. A two-tailed *p*-value < 0.05 was considered as statistically significant. Categorical variables were presented as frequencies (%). The normality of the distributions of the continuous variables was checked using the Shapiro–Wilk test. Continuous variables were reported as medians (quartile 1 (Q1) and quartile 3 (Q3)). As already mentioned, three slides from each block were evaluated, representing different depths, in order to eliminate potential differences between incisions arising from manual movement of the laser fiber. The median value between the three slides from each paraffin block was calculated for the incision depth, incision width and coagulation depth. The Kruskal–Wallis test was used to compare incision depth, incision width and coagulation depth between the three laser types. Post hoc Mann–Whitney U tests were performed for the three comparisons using a Bonferroni-adjusted alpha level of 0.0166 (0.05/3). Post hoc Mann–Whitney U tests were statistically significant if *p* < a level after Bonferroni correction for multiple comparisons, so if *p* < 0.0166. The carbonization effect (grade) was compared between the three laser technologies using Fisher’s exact test. The most frequent grade of each set of three slides was selected; if all grades (1, 2 and 3) were present, grade 2 was selected. The starting date of the study was 15 August 2025, while the end date was 15 September 2025, after finishing histopathological assessment and statistical analysis.

## 3. Results

**Figure 3:**
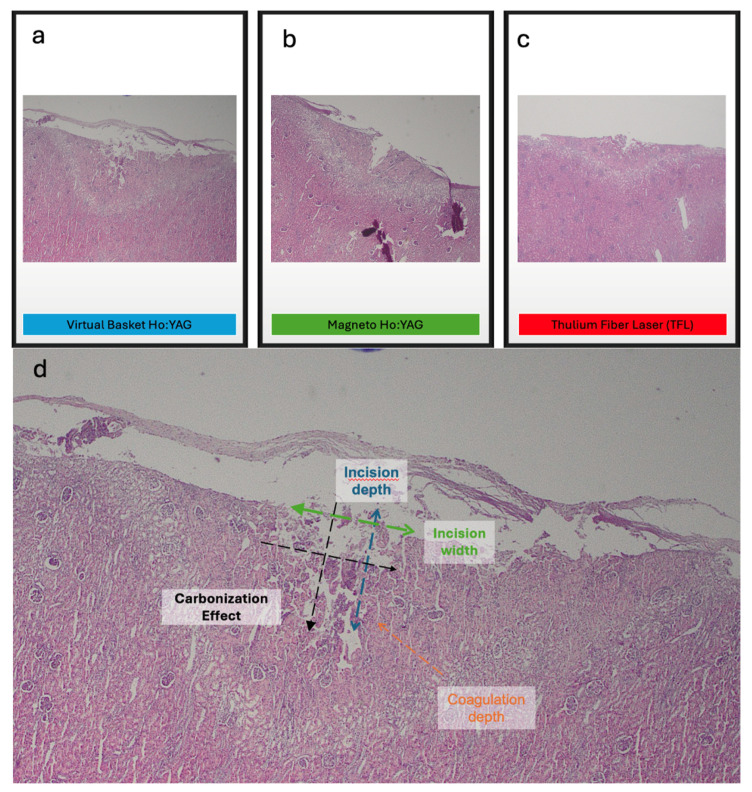
(**a**) The effect of VB Ho:YAG laser on soft tissue, (**b**) the effect of Magneto Ho:YAG laser on soft tissue, (**c**) the effect of TFL on soft tissue, and (**d**) pathological documentation of incision depth, incision width, coagulation depth and carbonization effect.

The impact of three different laser technologies on soft tissue is presented in Table 1. The median incision depth was 0.86 (0.53, 1.72) mm for the VB Ho:YAG laser, 0.60 (0.44, 0.83) mm for the Magneto Ho:YAG laser and 0.52 (0.24, 1.04) mm for the TFL (*p* = 0.213). The median incision width was 1.17 (1.04, 1.99) mm for the VB Ho:YAG laser, 1.05 (0.89, 1.50) mm for the Magneto Ho:YAG laser, and 0.82 (0.65, 0.88) mm for the TFL. These differences were statistically significant (*p* = 0.001). The median coagulation depth was 0.49 (0.41, 0.56) mm for the VB Ho:YAG laser, 0.51 (0.39, 0.59) mm for the Magneto Ho:YAG laser and 0.18 (0.17, 0.23) mm for the TFL. These differences were also found to be statistically significant (*p* < 0.001). The percentages of different grades of coagulation are presented in Table 1 and did not differ significantly between the three laser types. Figure 3 shows the effect of the three lasers on soft tissue (a, b, c) and explains the incision depth, incision width, coagulation depth and carbonization effect, as assessed by the two pathologists.

The different measurements of the laser-induced effects on kidney tissue are illustrated in this image using different colors:

Incision depth (blue): The maximum vertical distance from the tissue surface to the deepest point of the incision.

Incision width (green): The maximum horizontal extent of the incision, corresponding to the area of tissue loss.

Coagulation depth (orange): The zone of tissue between the incision and the adjacent normal tissue that appears altered by the laser; however, cytological and architectural features remain identifiable.

Carbonization effect (black): The region of tissue permanently altered by the thermal effect of the laser, characterized by non-viable tissue.

Post Hoc Mann–Whitney tests for the three comparisons revealed statistically significant differences only between the VB Ho:YAG laser and TFL and between the Magneto Ho:YAG laser and TFL for both the incision width and the coagulation depth (Table 2).

## 4. Discussion

The effect of different lasers on soft tissue ex vivo models have been previously assessed by other author groups. Doizi et al. evaluated the effect of the Ho:YAG laser and TFL in a porcine kidney model [21]. They reported that the Ho:YAG laser was associated with deeper incisions and better coagulation, while the TFL was associated with less fiber tip degradation [21]. However, the authors used different settings and equipment for their experiment. A 50 W TFL generator (IPG Photonics) was compared with a 120 W high-power Ho:YAG generator (P 120 H Lumenis, Israel) with no pulse modulation (non-Moses mode). The laser parameters (pulse energy and frequency) were standardized between the two devices and relevant for endoscopic enucleation of the prostate (EEP). For each parameter, different pulse durations (short, medium and long) were tested with the Ho:YAG generator, while different peak powers (150, 250 and 500 W) were tested with the TFL system. All incisions were performed with a 550 µm laser fiber, which was mounted on a robotic arm. The laser fiber was positioned 0.1 mm over the porcine kidney surface, moving with a fixed speed of 10 mm/s [17]. Interestingly, no carbonization zones were reported with the Ho:YAG laser, while they were prominent with the TFL and dependent on the peak power [21].

Likewise, Taraktin et al. compared the ablation, coagulation and carbonization characteristics between the Ho:YAG laser and TFL (both quasi-continuous and SuperPulsed) in non-frozen porcine kidneys [18]. They concluded that the SuperPulsed TFL produced comparable incisions with the Ho:YAG laser, while the quasi-continuous TFL was associated with rapid, deep and precise incisions and increased carbonization [22]. The equipment, laser settings and experimental setup were also different in their experiment. The authors reported using a 100 W Ho:YAG generator (Lumenis, Yokneam, Israel), a 120 W quasi-continuous TFL generator (NTO IRE-Polus, Fryazino, Russia/IPG Medical, Marlborough, MS, USA) and a 50 W SuperPulsed TFL generator (NTO IRE-Polus, Fryazino, Russia/IPG Medical, Marlborough, MS, USA). The settings were 40 W/1.5 J or 70 W/1.5 J for the Ho:YAG laser, 30 W/1.5 J or 60 W/1.5 J for quasi-continuous TFL and 30 W/1.5 J or 50 W/1.5 J for the SuperPulsed TFL. A 550 μm fiber was used for the Ho:YAG laser and a 600 μm fiber for the TFL. An XY Translation Stage was used in order to control the ablation speed [18]. It is worth mentioning that the authors observed an association between laser fiber speed and incision and coagulation depths [22].

Magneto was originally developed in an attempt to combine the benefits of the Ho:YAG laser and TFLs during endoscopic stone disease management. This is achieved by lowering the peak power of the Ho:YAG laser, producing longer pulse durations. These wavelength transformations are associated with better dusting capabilities and less stone retropulsion during laser lithotripsy [17,23]. In a recently published multicenter study, Perri et al. reported that the Magneto mode showed great ablation efficiency and low retropulsion rates during lithotripsy of both renal and ureteral stones [24]. These findings could potentially be translated to faster lithotripsy procedures and better stone-free rates (SFRs) [24]. However, the effect of the Magneto mode on soft tissue is still under investigation. Data of the Magneto mode during soft tissue surgeries currently arise from a single clinical study [14]. Perri et al. compared the 200 W Thulium:YAG Cyber TM generator (Quanta System) and the Ho:YAG (Cyber Ho generator with Magneto mode, Quanta System) lasers. The authors reported using 100 W for enucleation and 35 W for coagulation in the Thulium:YAG group, while in the Ho:YAG group they used 2 J/40 Hz in virtual basket mode for enucleation and 1 J/30 Hz in Magneto mode for early apical detachment and coagulation [14]. The authors concluded that enucleation with the Magneto mode was associated with better haemostasis, supported by the shorter morcellation time, increased morcellation efficiency and lower incident of hematuria in the Magneto group [14].

The ideal laser for performing EEP still represents a debatable issue. Uleri et al. performed a systematic review and meta-analysis comparing the Ho:YAG laser and TFL during EEP, reporting small differences between the outcomes of the two lasers [25]. The Ho:YAG laser was associated with shorter enucleation time and better Qmax at three months postoperatively, while the TFL was associated with better IPSS at three months postoperatively and better Qmax at 6–12 months postoperatively [25]. Nevertheless, complication rates were comparable between the two groups [25]. Soft tissue surgery is not limited to EEP, with endoscopic management of UTUC gaining increasing popularity in recent years, especially for low-risk patients. Taraktin et al. investigated whether the outcomes of kidney-sparing surgery could be dependent on laser selection [6]. They concluded that currently there is not enough evidence to defend the superiority of one laser over the other, while surgeon’s experience and preference remain the most defining factors for selecting the most appropriate laser for endoscopic management of UTUCs [6].

This ex vivo study has several limitations. To begin with, only two porcine kidneys were used, and no power analysis was conducted. However, this was a systematic study under reproducible conditions and the inclusion of additional renal units and animals would oppose with ethical standards of animal experiments. Moreover, both surfaces of the two kidneys were used, enabling multiple laser incisions with the different laser technologies. Another important limitation was the absence of a standardized stabilization system, such as a robotic arm, which could ensure constant speed of the laser fiber and distance from the kidney surface. Nevertheless, several steps were followed in order to address these limitations. All laser incisions were performed by the same surgeon using a standardized technique, with each laser line being applied for a predefined duration of three seconds. At the end of the procedure, all incisions were reviewed for uniformity and only 12 out of the 16 initially marked areas, those with the most uniform lines, were included in the final analysis. Furthermore, three slides from each block were evaluated, representing different depths, to eliminate potential differences between incisions arising from manual movement of the laser fiber. The median value between the three slides from each paraffin block was calculated for the incision depth, incision width and coagulation depth, while the most frequent grade of each set of three slides was selected. An important limitation is also the ex vivo design itself. Although the choice of the porcine kidney is classically used, the inherent limitations of the ex vivo model, including the absence of perfusion, actual bleeding and healing, must be recognized. However, in an attempt to preserve perfusion and viable vessels, we kept an interval of less than 30 min between tissue fixation and ligation of the two renal pedicles. Optical interactions, such as bubble formation in saline, were not quantified in the current study, while tissue degradation effects were not assessed. Additionally, coagulation depth was defined as the deepest clotted vessel and was not validated against quantitative thermal imaging or optical coherence tomography, while the carbonization effect was assessed using a bespoke scale, defined by the pathologists. Moreover, the exact peak powers of the virtual basket Ho:YAG laser and TFL were not provided, because they are not officially reported by the manufacturers. Another limitation can be found to the pathologists’ ratings. Carbonization grading was performed independently by two blinded pathologists, followed by a consensus meeting of the pathologists’ ratings, during which discrepancies were resolved, and a final adjudicated grade was assigned. The dataset, therefore, contained consensus ratings rather than independent rater scores, and inter-rater agreement statistics (e.g., weighted kappa for the ordinal carbonization grade) were not computed. Finally, the laser settings that we used were lower than those reported in other ex vivo studies comparing the effect of different laser technologies on soft tissue. These settings were selected to ensure operator safety and to reflect the broader spectrum of lasers on urological soft tissue surgery, which extends beyond EEP, including endoscopic management of UTUCs. Nevertheless, safety parameters, such as fiber degradation and soft tissue retropulsion, were not assessed in the current study. The same energy settings and laser fiber diameters were used for performing incisions with all three lasers. Future research should focus on whether the different effects of the three lasers on soft tissue depend on energy settings. It thus remains questionable whether differences between the three lasers reported in this experiment are preserved when higher-energy settings are applied. Unlike some other ex vivo experiments, pulse modulation mode was employed for the conventional Ho:YAG laser incisions in virtual basket mode. This methodological peculiarity may justify potential discrepancies between our study and the literature.

## 5. Conclusions

Both VB and Magneto Ho:YAG lasers produced laser incisions with statistically significant greater incision width and coagulation depth than the TFL on the ex vivo model. Overall, the Magneto Ho:YAG laser was associated with the greatest median coagulation depth. Post Hoc Mann–Whitney tests for the three comparisons revealed statistically significant differences only between the VB Ho:YAG laser and TFL and between the Magneto Ho:YAG laser and TFL. This finding could potentially be translated into better haemostasis during endourological soft tissue surgery. The implementation of additional studies, both experimental and clinical ones, is of outmost importance to draw safer conclusions. Moreover, future research should focus on whether the different effects of the three lasers on soft tissue depend on energy settings.

## Figures and Tables

**Table 1:** The median incision depth, incision width, coagulation depth and the dominant grade for all three laser technologies.

Outcome/Laser		VB	MH	TFL	*p*-Value
**Incision Depth (mm)**		0.86 (0.53, 1.72)	0.60 (0.44, 0.83)	0.52 (0.24, 1.04)	0.213 *
**Incision Width (mm)**		1.17 (1.04, 1.99)	1.05 (0.89, 1.50)	0.82 (0.65, 0.88)	0.001 *
**Coagulation Depth (mm)**		0.49 (0.41, 0.56)	0.51 (0.39, 0.59)	0.18 (0.17, 0.23)	<0.001 *
**Grade**	**Grade 1**	4 (33.3%)	6 (50.0%)	7 (58.4%)	0.833 **
	**Grade 2**	6 (50.0%)	4 (33.3%)	4 (33.3%)	
	**Grade 3**	2 (16.7%)	2 (16.7%)	1 (8.3%)	

* Kruskal–Wallis test; ** Fisher’s exact test (the most frequent grade was selected; if all grades, 1, 2 and 3, were present: grade 2 was selected).

**Table 2:** Post hoc Mann–Whitney U tests for the three comparisons using a Bonferroni-adjusted alpha level of 0.0166 (0.05/3).

Comparison/Outcome	Incision Width (mm)	Coagulation Depth (mm)
**VB vs. MH**	0.166	0.707
**VB vs. TFL**	**<0.001**	**<0.001**
**MH vs. TFL**	**0.009**	**<0.001**

Post hoc Mann–Whitney U tests—statistically significant if *p* < a level after Bonferroni correction for multiple comparisons: so, if *p* < 0.0166.

## Data Availability

All data are available on request by the corresponding author.

## Supplementary Materials

The following supporting information can be downloaded at: https://www.mdpi.com/article/10.3390/jcm15031074/s1, The ARRIVE guidelines 2.0: author checklist.

## Abbreviations

The following abbreviations are used in this manuscript:

Ho:YAG	Holmium:YAG
TFL	Thulium Fiber Laser
VB	Virtual Basket
BPO	Benign Prostatic Obstruction
UTUC	Upper Tract Urothelial Carcinoma
UPJO	Ureteropelvic Junction Obstruction
HoLEP	Holmium Laser Enucleation of the Prostate
EEP	Endoscopic Enucleation of the Prostate
SFRs	Stone-Free Rates
